# Continuous Regional Arterial Infusion with Fluorouracil and Octreotide Attenuates Severe Acute Pancreatitis in a Canine Model

**DOI:** 10.1371/journal.pone.0037347

**Published:** 2012-05-24

**Authors:** Meng Tao Zhou, Bi Cheng Chen, Hong Wei Sun, Yue Peng Jin, Fa Jing Yang, Xing Zhang, Roland Andersson, Qi Yu Zhang

**Affiliations:** 1 Department of Surgery, The First Affiliated Hospital, Wenzhou Medical College, Wenzhou, Zhejiang Province, China; 2 Zhejiang Provincial Top Key Discipline in Surgery, Wenzhou Key Laboratory of Surgery, Department of Surgery, The First Affiliated Hospital, Wenzhou Medical College, Wenzhou, Zhejaing Province, China; 3 Department of Surgery, Clinical Sciences Lund, Lund University and Skane University Hospital, Lund, Sweden; University of Valencia, Spain

## Abstract

**Aim:**

To investigate the therapeutic effects of fluorouracil (5-Fu) and octreotide (Oct) continuous regional arterial infusion (CRAI,) alone or in combination, was administered in a canine model of severe acute pancreatitis (SAP).

**Materials and Methods:**

The animals were divided into five groups; group A (Sham), group B (SAP), group C (SAP and 5-Fu), group D (SAP and Oct), and group E (SAP and 5-Fu + Oct). Levels of amylase, α-tumor necrosis factor (TNF-α), blood urea nitrogen (BUN), creatinine, thromboxane B2 and 6-keto- prostaglandin F1α were measured both before and after the induction of SAP. Pathologic examination of the pancreas and kidneys was performed after termination of the study.

**Results:**

Pathologic changes noted in the pancreas in SAP significantly improved following CRAI with either single or combined administration of 5-Fu and Oct, where combination therapy demonstrated the lowest injury score. All treatment groups had significantly lower levels of serum TNF-α and amylase activity (*P*<0.05), though only groups D and E had a lower BUN level as compared to group B. The plasma thromboxane B_2_ level increased in SAP, but the ratio of thromboxane B_2_/6-keto- prostaglandin F_1α_ decreased in the treatment groups, with the combination therapy (group E) demonstrating the lowest ratio as compared to the other 3 experimental groups (*P*<0.05).

**Conclusions:**

The findings in the present study demonstrate an attenuation of SAP in a canine model following CRAI administration with 5-Fu or Oct, alone or in combination.

## Introduction

Despite advances in intensive care, the mortality rate of severe acute pancreatitis (SAP) still remains high, ranging from 10 up to 25% [1]. In its severe form, activation of oxygen-free radicals and microcirculatory changes, with increased endothelial barrier permeability, may result in a profound systemic inflammatory response, development of remote organ injury, potentially culminating in the multiple organ dysfunction syndrome (MODS) [2]–[6].

The inflammation and necrosis within the pancreas is a triggering factor for the development of MODS, and thus resolution of these local events is crucial. However, compounds with a property of suppression of the severe inflammation in pancreas are rarely reported. By inhibiting pancreatic secretion, somatostatin and its analogues, which have been reported to indirectly reduce MPO activity [7], release of inflammatory mediators [8], prevention of bacterial translocation [9] and ischemia-reperfusion injury [10], have been used in SAP patients [11]. Also the cytotoxic drug 5-fluorouracil (5-Fu) has been reported to alleviate the pancreatic injury by inhibiting the inflammatory response and auto-digestion of the pancreas during the early stage of disease [12], [13].

However, as the half-life of somatostatins is short and the inhibitory effect on trypsin secretion is dose-dependent, the relevance of somatostatin treatment in SAP has been questioned. Furthermore, 5-Fu as a cytotoxic drug used for treating a non-malignant disease still is not fully acceptable in the western world. Continuous regional arterial infusion (CRAI) may be a useful mode of administration in order to resolve the problems, and CRAI has been reported as being an effective way of drug delivery also in acute pancreatitis to obtain high drug concentrations within the pancreas with minimal systemic toxic effects [14].

The effects of CRAI with Oct or 5-Fu, alone or in combination, has not been studied. The purpose of the present study was thus to assess the potential therapeutic effect of CRAI with administration of octreotide and 5-Fu in a canine model of SAP.

## Materials and Methods

### Animals

Nineteen adult dogs of both sexes, weighing between 10 and 13 kg, were used. The animals were supplied by the Laboratory Animal Center of Wenzhou Medical College (Wenzhou, China), kept under conventional conditions, and fed an ordinary laboratory diet and water. All experiments were performed in animals after 12 h fasting with free access to water.

### Ethics Statement

The protocol for the animal experiment was approved by the Institutional Animal Committee of Wenzhou Medical College. All animals received care in accordance to the ‘Guide for the Care and Use of Laboratory Animals’. Procedures using dogs were approved by the Institutional Animal Care and Use Committee of Wenzhou Medical College (document number: wydw2011-0001).

### Experimental Protocol

The animals were randomly divided into five experimental groups; sham group (group A; n = 5) and 4 groups with SAP: Group B (CRAI with physiological saline; n = 4), group C (CRAI with 5-Fu infusion; n = 5), group D (CRAI with administration of Oct; n = 5), and group E (CRAI with 5-Fu and Oct; n = 5).

### Induction of Severe Acute Pancreatitis

All procedures were performed under sterile conditions. The adult mongrel dogs were anesthetized by intravenous injection of 1.5% pentobarbital sodium (1.5 ml/kg, Beijing Chemical Reagent Company, Beijing, China). The right femoral vein was cannulated with a 16-gauge intravenous hyperalimentation catheter to infuse lactated Ringer’s solution at a rate of 10 ml/kg/h. The right femoral artery was cannulated with a 20-gauge Teflon catheter for blood sampling. Thirty minutes before the operation, 0.5% metronidazole injection (50 ml) and 500 mg cephalosporin were injected in all animals to prevent infection. After an upper abdominal midline incision, the right gastroepiploic artery was cannulated with an epidural catheter. The tip of the catheter was placed in the gastroduodenal artery near the pylorus, the level confirmed by injection of methylene blue, staining the whole of the pancreas. According to Aho et al [15], severe acute pancreatitis was induced by the retrograde intraductal injection of 5% sodium taurocholate (1 mL/kg; Sigma Chemical Company, St Louis, MO, USA ) to the main pancreatic duct through a microinjection pump at a speed of 2 mL/min. The sham group had the cannulation procedure, though without the 5% sodium taurocholate injection.

### Continuous Regional Arterial Infusion Therapy

Parallel with the retrograde intraductal injection of 5% sodium taurocholate, the CRAI therapy was initiated. The CRAI perfusate ingredients were as follows: group A (sham) and group B (SAP) had infusion of physiological saline (2 ml/h), and in groups C, D and E, the drugs were diluted in 50 ml physiological saline and infused at a speed of 2 ml/h with 5-Fu (15 mg/kg; Nantong General Pharmaceutical Factory, Nantong, Jiangsu, China), octreotide (10 µg/kg; Novartis Pharmaceutical Co., Switzerland) or the combination of both drugs. The dose of both drugs used in present study was the one corresponding to what has been used in routine clinical practice in China.

Rehydration and electrolyte balance was kept during the perioperative course.

### Collection of Specimens

Three microliters of blood was withdrawn from the right femoral artery at the time of abdominal incision, at the injection of saline or 5% sodium taurocholate, and 15min, 1 h, 3 h, 6 h, 10 h after the induction of SAP. Serum was stored at −80^o^C. All dogs in the sham group were sacrificed at 36 h. Immediately after sacrifice (by blood-letting from the abdominal aorta, the pancreas and kidneys in the sham group were removed and subsequently fixed with 10% formaldehyde. Corresponding samples from the 4 experimental SAP groups were collected from the pancreas and kidneys immediately after death from SAP progression.

**Table 1:** Survival time in the various groups.

Group	Survival time					*P* Value		
						Group B *vs.*	Group C *vs.*	Group D *vs.*
Group B (n = 4)	12 h 35 min	13 h 55 min	16 h 3 min	18 h 45 min				
Group C (n = 4)	23 h 29min	12 h 25 min	17 h 12 min	19 h 3 min		0.377		
Group D (n = 5)	27 h 58 min	19 h 4 min	35 h	22 h 25 min	14 h 35 min	0.020	0.119	
Group E (n = 5)	27 h 1 min	30 h 20 min	23 h 56 min	19 h 3 min	36 h 5 min	0.003	0.022	0.347

Group B  =  SAP  =  severe acute pancreatitis.

Group C  =  SAP + CRAI with 5-FU.

Group D  =  SAP + CRAI with octreotide.

Group E  =  SAP + CRAI with 5-FU and octreotide.

*P* value, the comparison of the statistical value between every two group.

### Cytokines and Biochemical Parameters

Serum amylase levels was determined by means of iodine-amylum colorimetry and expressed in u/dl (Ningbo Cicheng Biotechnology Reagent Factory, Ningbo, Zhejiang, China). Serum levels of TNF-α were determined by enzyme-linked immunosorbent assay (ELISA; Rapidbio Company, USA) and expressed in ng/L. Serum urea nitrogen was determined by urease-GLDH and expressed in mmol/L (Shanghai Changzheng Biochemical Reagent CO., LTD, Shanghai, China). Serum creatinine was analyzed by Jaffe’s Kinetic and expressed in µmol/L and serum TXB_2_, 6-k-PGF_1_ by radioimmunoassay and expressed in ng/L (Suzhou Medical College, Suzhou, China). All parameters were determined by using commercial kits and conducted according to the protocols provided by the manufacturers.

### Pathologic Analysis and Grading Criteria

Autopsy was immediately carried out after sacrifice or death and the severity of gross lesions were scored and graded. Scoring was based on pathologic observations as reported by Prof. Zhang Shengdao [16]. In brief, the pancreas was cut to three sections (head, body and tail), after which each section was divided into four equal portions (totally 12 portions). The peripancreatic tissues were divided into the great omentum and the mesentery between the pancreas and duodenum (3 portions). The appearance of haemorrhage and necrotic lesions in each portion were noted. Totally 15 portions of tissue were harvested and observed, the points recorded and added for evaluation. According to the total score, the pancreas gross lesion severity was divided into four grades: grade I: ≤5 points; grade II: 6–9 points; grade III: 10–13 points; grade IV: ≥14 points.

Pancreas and kidney specimens were harvested and fixed in 10% formaldehyde solution, embedded in paraffin, sectioned, and stained with hematoxylin- eosin (H&E) for light microscopy. The pathologic grading were scored as described by Schmidt et al [17], including pancreatic edema, hemorrhage, putrescence, inflammation, as well as peripancreatic fat necrosis and calcification. The pathologic changes in the kidneys were analyzed and classified according to a previously described acute renal injury score [18]. The sections were examined by an experienced pathologist in a blinded fashion.

### Statistical Analysis

The data were analyzed by SPSS software (11.5 version). Statistical significance of differences among multiple groups was determined by one-way ANOVA (when the variance was even) or rank-sum test (when the variance was uneven). Multiple comparisons were determined by SNK-q test (when the variance was even) or Nemenyi test (when the variance was uneven). For ranked data, statistical significance for differences was determined by Ridit test. For all analyses, statistical significance was defined as *p*<0.05.

**Figure 1:**
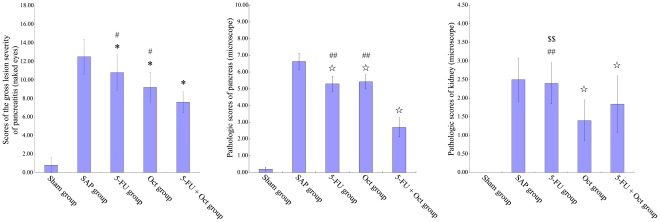
Pathological scores of the pancreas and kidneys following CRAI with 5-Fu and Octreotide (Oct). Three treatment groups have lower pancreatic pathologic scores than SAP group. Combination using of 5-Fu and Oct significantly improved the pancreatic pathologic scores as compared to the single use of 5-Fu or Oct. Both Oct treatment groups demonstrated lower pathologic scores than the 5-Fu group. No significant pathologic changes were found in the sham group. *: vs. SAP group, P<0.05; ⋆: vs. SAP group, P<0.01; $: 5-FU group vs. Oct group, P<0.05; $$: 5-FU group vs. Oct group, P<0.01; #: 5-FU group or Oct group vs. 5-FU + Oct group, P<0.05; ##: 5-FU group or Oct group vs. 5-FU + Oct group, P<0.01.

## Results

### Survival Time of SAP Dogs

After establishment of the SAP model, the dogs were under anesthesia during the whole study period. All SAP dogs in groups B and group C died within 24 h, though in groups D and group E, two dogs survived after 24 h (Table 1). All dogs in the sham group survived until sacrificed after 36 h post-operation.

### Pathologic Examination of the Pancreas and Kidneys

At the autopsy examination, pathologic examination was normal in group A (sham). Typical SAP features were found in group B, including patchy putrescent with saponified spots and color changes, and hemorrhagic ascites in the abdominal cavity. In groups C, D and E, the changes were attenuated as compared to group B. Among the different interventional groups, group E (5-FU + Oct) showed significantly less changes (Figure 1).

According to the method described by Schmidt et al [17], the scores in groups C and D were similar, both groups having significantly lower scores as compared to group B (*P<0.01*). The pathologic scores of group E were the lowest among all experimental groups (*P<0.01*; Figure 1).

As comes the histological examination of the kidneys, group B showed obvious congestion of the glomerular capillaries, swelling of the renal tubular epithelial cells, scattered or lamellar necrosis, protein cast, interstitial edema and inflammatory cell infiltration [18]. In group C, the pathologic changes and score were similar to group B. There was no significant difference in pathologic scores between groups D and E, while both groups had lower scores than groups B and C (*P<0.05*; Figure 1).

**Figure 2:**
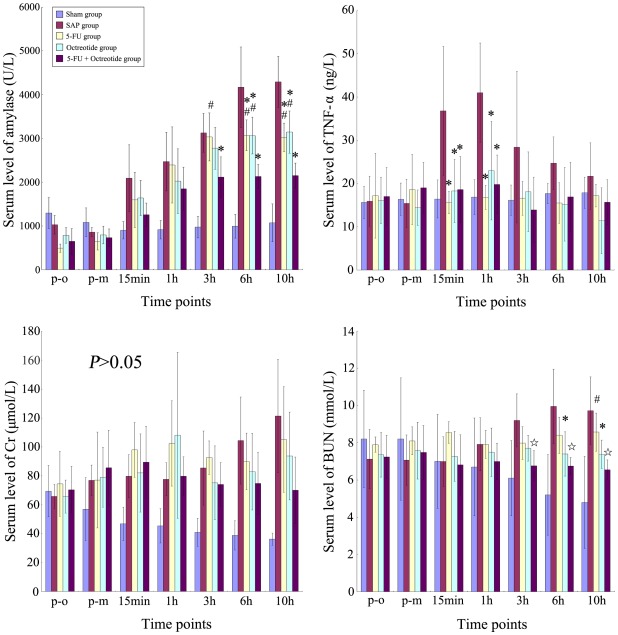
CRAI with 5-Fu and Octreotide (Oct) attenuated serum parameters of pancreatitis. Three treatment groups have lower serum levels of amylase activity, TNF-α and BUN as compared to the SAP group at different time points. The combined use of 5-Fu and Oct significantly decreased the levels of amylase activity as compared to single use of 5-Fu or Oct. Both Oct treatment groups had a lower BUN level as compared to the SAP group. No significant changes of serum levels of amylase activity, TNF-α, Cr and BUN were found in the sham group. *: vs. SAP group, P<0.05; ⋆: vs. SAP group, P<0.01; $: 5-FU group vs. Oct group, P<0.05; $$: 5-FU group vs. Oct group, P<0.01; #: 5-FU group or Oct group vs. 5-FU + Oct group, P<0.05; ##: 5-FU group or Oct group vs. 5-FU + Oct group, P<0.01.

**Figure 3:**
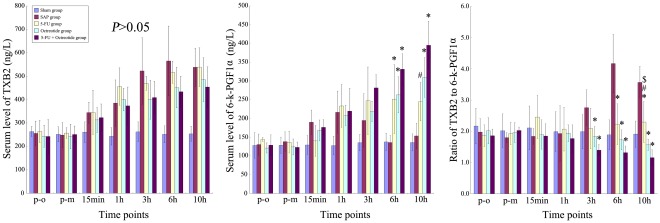
CRAI with 5-Fu and Octreotide (Oct) increased the ratio of TXB_2_ to 6-k-PGF_1α._ No significant difference of TXB_2_ was found among the 4 experimental groups. However, 6-k-PGF1α significantly increased in all 3 treatment groups. So the ratio of TXB2 to 6-k-PGF1α was significantly lower in the treatment groups as compared to the SAP group. The sham group had normal TXB2, 6-k-PGF1α, and ratio of TXB2 to 6-k-PGF1α. *: vs. SAP group, P<0.05; ⋆: vs. SAP group, P<0.01; $: 5-FU group vs. Oct group, P<0.05; $$: 5-FU group vs. Oct group, P<0.01; #: 5-FU group or Oct group vs. 5-FU + Oct group, P<0.05; ##: 5-FU group or Oct group vs. 5-FU + Oct group, P<0.01.

### Serum Amylase Levels

There was no significant difference between parameters when comparing the different groups before SAP was initiated. Serum amylase levels gradually increased in all groups following induction of SAP. In groups C and D, the amylase levels were significantly lower than in group B at 6 and 10 hrs following SAP induction (*P<0.05*). In group E, serum amylase levels significantly decreased from 3 h and on as compared with group B (*P<0.05*). In addition, serum amylase levels in group E was significantly lower as compared to group C at 3, 6, and 10 h and to group D at 6 and 10 h (*P<0.05;* Figure 2).

### Serum TNF-α Levels

Following the establishment of SAP, serum TNF-α levels gradually increased, reaching a peak at 1 h and then declined. All test groups had the same pattern of TNF-α changes, but TNF-α levels in the treated groups were significantly lower as compared to group B at 15min and 1 h (*P<0.05*). No significant differences were found among the experimental groups at the other time points, before and after SAP-induction (*P<0.05;* Figure 2).

### Serum Levels of Urea Nitrogen and Creatinine

Serum levels of urea nitrogen and creatinine gradually increased within the first 10 h process in group B. No significant differences were found between groups B and C at all time points studied. In group D, urea nitrogen levels was significantly lower as compared to group B at 6 and 10 h (*P<0.05*), but creatinine levels did not significantly differ between groups B and C at all time points studied. In group E, urea nitrogen levels was significantly lower as compared to group B at 3, 6 and 10 h (*P<0.01*), but creatinine levels did not significantly differ between groups D and E at all time points. Serum levels of urea nitrogen in group E was significantly lower as compared to group C at 10 h (*P<0.05;* Figure 2).

### Serum Levels of TXB_2_, 6-k-PGF_1α_ and the Ratio of TXB_2_ to 6-k-PGF_1α_

Serum level of TXB_2_ significantly rose after SAP induction in all four groups, but no significant differences were found among the four groups at each time point. In group C, 6-k-PGF_1α_ levels were significantly higher as compared to group B at 6 hrs (*P<0.05*). In groups D and group E, 6-k-PGF_1α_ levels were significantly higher as compared to group B at 6 and 10 h (*P<0.05*). In addition, serum level of 6-k-PGF_1α_ in group E was significantly higher as compared to group C at 10 h (*P<0.05;* Figure 3).

In group C, the ratio of TXB_2_ to 6-k-PGF_1α_ was significantly lower as compared to group B at 6 and 10 h (*P<0.05*). In groups D and E, the ratio was significantly lower as compared to group B at 3, 6 and 10 h (*P<0.05*). In addition, the ratio in group D was significantly lower as compared to group C at 10 h and the ratio in group E was significantly higher as compared to group C at 10 h (*P<0.05;* Figure 3).

## Discussion

Due to the systemic release of pancreatic enzymes and cytokines, SAP can affect remote organs. As a main initiation factor of ARDS, the local inflammation within the pancreas plays a crucial role in the progression of SAP. However, the effect of CRAI with 5-Fu or/and Oct has not been known. In the present study, a canine SAP model was developed, confirmed e.g. by pathologic changes and an increase in serum levels of amylase activity and TNF-α. CRAI with 5-Fu or/and Oct ameliorated the SAP, including the otherwise occurring pathologic changes, and increases in serum amylase and TNF-α levels. Furthermore, a systemic protective effect provided by CRAI was noted, evidenced by decreased levels of serum TNF-α, the ratio of TXB_2_ to 6-k-PGF_1α_ and urea nitrogen and lower scores at renal pathologic evaluation.

Octreotide has for long been recommended in SAP treatment, though the actual effects have been discussed [19]. Octreotide in the treatment of SAP is dose-dependent and the effect could be limited by the blood-pancreatic tissue interface, e.g. by impaired microcirculation and ischemia [20]–[22]. CRAI has been proven to dramatically increase the drug concentrations within the pancreas [23], [24]. In our study, CRAI with Oct in experimental SAP significantly decreased the release of amylase into peripheral blood and improved the effect on both the local and systemic inflammatory response. The results confirm that the beneficial effects of octretide are achieved locally within the pancreas. The systemic effect should be indirect, as evidenced by a decreased systemic inflammatory response and less endothelial injury, as previous studies have demonstrated a decrease in endotoxin generation, inhibition of the release of inflammatory mediators and platelet aggregation, protection of the liver and a decrease in ischemia-reperfusion injury [9], [10], [25]–[27]. CRAI provides a significant increase in protease inhibitor concentrations in pancreatic tissues, as compared to a corresponding intravenous injection, and trypsin levels are significantly suppressed [28], [29]. Though a randomized controlled trial (RCT) has not been conducted, the benefit of CRAI has been documented [30], [31].

5-Fluorouracil (5-Fu) has been tried in acute pancreatitis since the 1970s [32], [33]. Essentially, 5-Fu can decrease the synthesis of pancreatic enzymes, or serve as a proteinase inhibitor [34], [35]. In the present study, CRAI with 5-Fu decreased the serum amylase levels. Thus, it is thought to alleviate the pancreatic injury through prevention of auto-digestion of the pancreas during the initial stage of SAP [36]. Several studies in experimental pancreatitis have shown promising results with 5-Fu treatment, such as affecting amylase, trypsin and survival rates [32], [37]. Chen *et al* confirmed that 5-Fu modulated the pro-inflammatory cytokine response in experimental acute pancreatitis [38]. Clinical studies have documented that treatment with 5-Fu can reduce the mortality and hospital stay [39], [40]. In China, the administration of 5-Fu has been considered as an adjuvant therapy for SAP, and been reported associated with beneficial results [12], [41].

We postulated that the combined use of the two drugs, administered via CRAI, would achieve a synergetic effect. The beneficial effects of the two drugs could depend upon the different mechanisms provided by 5-Fu and octreotide, respectively. 5-Fu mainly attenuates the local inflammation within the pancreas and octreotide inhibits the auto-digestive effect. In this study, we found that the CRAI with combined 5-Fu and Oct treatment showed the best result of the studied parameters. Comparing CRAI with 5-Fu or Oct, CRAI with 5-Fu and Oct together showed a less degree of vacuolization, tissue edema, inflammatory cell infiltration, hemorrhage and necrosis within the pancreas, as well as less renal pathologic changes and reduced urea nitrogen and creatinine. In SAP, TNF-α effects endothelial cells, evokes the disturbances of pancreatic microcirculation and dysfunction of remote organs. Significant changes of peripheral concentrations of Thromboxane A2 (TXA2) and PGI2 represent mass synthesis of both factors by injured endothelial cells. TXA2 and PGI2 are precursor of thromboxane B2 (TXB2) and 6-keto-prostaglandin-F1-alpha (6-k-PGF1alpha), respectively, and participate in endothelial microvascular injury. Consistent with a previous study [41], a decreased TXB2/6-K-PGF1α (TXA2/PGI2) ratio was related to less pathologic changes of the pancreas. Reduced endothelial injury, reflected by a decreased TXA2/PGI2 ratio, was found following CRAI with 5-Fu and Oct, which is a potential reason for the improvement of SAP-associated renal injury.

The main adverse occurrence in the early phase of SAP, i.e. the systemic inflammatory response syndrome (SIRS), the main driving factor for the development of the acute respiratory distress syndrome (ARDS), the dominant component of multiple organ dysfunction syndrome (MODS) and the main cause of death [42], might be possible to overcome by the early use of CRAI with 5-Fu and Oct, though this of course warrants further studies in order to verify the potential effect and clarify the mechanisms involved.
