# Correction: Continuous Regional Arterial Infusion with Fluorouracil and Octreotide Attenuates Severe Acute Pancreatitis in a Canine Model

**DOI:** 10.1371/annotation/9e189146-9c58-44d0-8bff-347bde7018e9

**Published:** 2013-10-08

**Authors:** Meng Tao Zhou, Bi Cheng Chen, Hong Wei Sun, Yue Peng Jin, Fa Jing Yang, Xing Zhang, Roland Andersson, Qi Yu Zhang

There was an error in the Funding statement. The correct version of this Funding statement is available below.

This study was sponsored by grants of Zhejiang Provincial Top Key Discipline in Surgery, the Natural Science Foundation of Zhejiang Province (Grant No. Z2100853) and the National Natural Science Foundation of China (Grant No. 81070372). The funders had no role in study design, data collection and analysis, decision to publish, or preparation of the manuscript. 

